# Data demonstrating the *in vivo* anti-tumor efficacy of thermosensitive liposome formulations of a drug combination in pre-clinical models of breast cancer

**DOI:** 10.1016/j.dib.2023.109545

**Published:** 2023-09-09

**Authors:** Xuehan Wang, Maximilian Regenold, Michael Dunne, Pauric Bannigan, Christine Allen

**Affiliations:** Leslie Dan Faculty of Pharmacy, University of Toronto, 144 College Street, Toronto, Ontario, M5S 3M2, Canada

**Keywords:** Thermosensitive liposomes, Drug delivery, Hyperthermia, Doxorubicin, Alvespimycin, Triggered drug release, Nanomedicine

## Abstract

Thermosensitive liposomes in combination with localized mild hyperthermia can improve the delivery of drug to solid tumor sites. For this reason, thermosensitive liposome formulations of a range of chemotherapy drugs have been designed. Our group previously developed and characterized a thermosensitive liposome formulation of the heat shock protein 90 inhibitor alvespimycin as a companion therapeutic to a thermosensitive liposome formulation equivalent in composition to ThermoDox (i.e., ThermoDXR), with the goal of increasing the therapeutic index of doxorubicin as the combination was revealed to be highly synergistic in a panel of human breast cancer cell lines including MDA-MB-231 (Dunne et al., 2019). The data presented here further describes the effect of the doxorubicin (DXR) and alvespimycin (ALV) combination *in vitro* and *in vivo*. Specifically, the combination effect in mouse breast cancer 4T1 cells and the *in vivo* efficacy of this heat-activated chemotherapy combination in both immunocompromised (MDA-MB-231 tumor bearing female SCID mice) and immunocompetent (4T1 tumor bearing female BALB/c mice) models of breast cancer.

## Graphical Abstract

**Figure:**
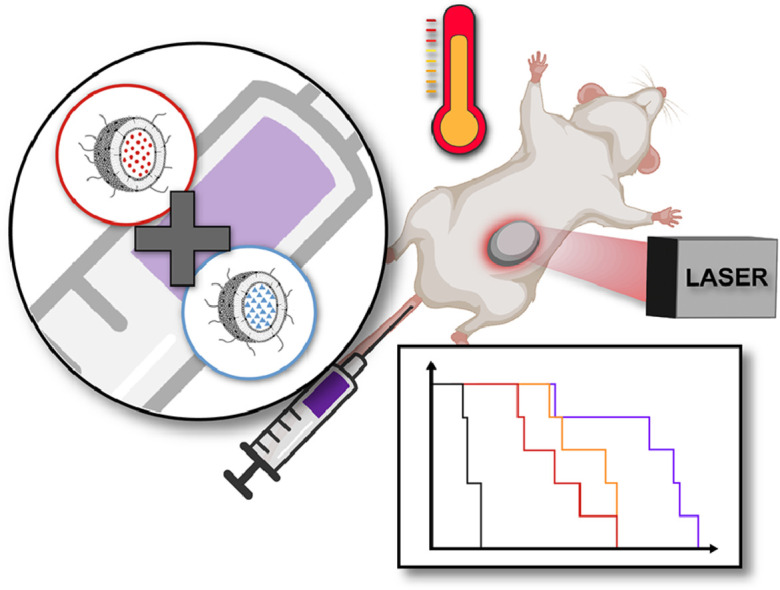

Specifications Table

**Table:** 

Subject	Oncology; Nanotechnology.
Specific subject area	Evaluation of treatment efficacy of a thermosensitive liposome combination and localized mild hyperthermia in two breast cancer models in mice.
Type of data	Graph Table
How the data were acquired	The half maximal inhibitory concentrations (IC_50_) of doxorubicin and alvespimycin monotherapy and combinations were determined using the acid phosphatase (APH) assay. CalcuSyn Version 2.0 was used to calculate the combination indices based on the collected IC_50_ values. Tumor length (l) and tumor width (w) were measured using 15 cm calipers every other day for the duration of the study. Tumor volumes were calculated using the following equation $V=\;\frac{\pi }{6}\left(w^{2}\right)\left(l\right)$. Animal weights were recorded using a digital mass balance every other day for the duration of the study. Both tumor volumes and animal weights were expressed as percentages of initial measurements. Statistical analysis of tumor growth and animal body weight was performed using SSPS Statistics 28.0. Tumor dimensions of > 15 mm in any direction, or weight loss > 20 % initial body mass, were selected as ethical endpoints. Kaplan-Meier survival analysis for both animal models was performed using GraphPad Prism 9.3.0.
Data format	Raw Analyzed Filtered
Description of data collection	4T1 cells were treated with DXR and/or ALV at either 37 or 42 °C. 2 mg/mL *p*-nitrophenylphosphate were incubated with the cells prior to the measurement of UV absorbance at 405 nm. Tumor volume, animal weight, and survival data were measured every other day following treatment of mice with thermosensitive liposome formulations in combination with mild hyperthermia.
Data source location	Leslie Dan Faculty of Pharmacy, University of Toronto 144 College Street, Toronto, Ontario, M5S 3M2, Canada
Data accessibility	Repository name: Mendeley Data Data identification number: 10.17632/b4b86vtsgx.1 Direct URL to data: https://data.mendeley.com/datasets/b4b86vtsgx/1
Related research article	M. Dunne, B. Epp-Ducharme, A.M. Sofias, M. Regenold, D.N. Dubins, C. Allen, Heat-activated drug delivery increases tumor accumulation of synergistic chemotherapies, J. Controlled Release. 308 (2019) 197–208. https://doi.org/10.1016/j.jconrel.2019.06.012.

## 1 Value of the Data

• The data describe the therapeutic efficacy of a drug combination delivered in thermosensitive liposomes in two preclinical breast cancer models. Similar thermosensitive liposome formulations have been shown to improve drug accumulation within tumours following triggered release using mild hyperthermia [1]. These data further demonstrate the promising potential of delivering drug combinations in thermosensitive liposomes for applications in oncology.
• These data demonstrate that combination therapy affords similar therapeutic efficacy to high dose doxorubicin while simultaneously reducing systemic toxicity. These findings are relevant to research communities focused on drug delivery, nanotechnology, and oncology. In particular, for those interested in thermosensitive nanomedicines of chemotherapy or formulation strategies for the delivery of heat shock protein inhibitors.
• These data may be used by investigators to inform the design of new and improved thermosensitive liposome formulations of chemotherapeutic drugs. The data demonstrate the *in vivo* efficacy and toxicity of this treatment approach and thus contribute to the understanding of thermosensitive liposome mediated combination chemotherapy.

## 2 Objective

A synergistic drug combination of DXR and ALV was previously identified and formulated into thermosensitive nanocarriers to improve tumor targeting [1]. We continue to evaluate the therapeutic effect and systemic toxicity of the thermosensitive liposome combination in conjunction with mild hyperthermia treatment using two commonly used preclinical breast cancer models. This dataset provides a more comprehensive understanding of the *in vivo* performance of the proposed treatment approach.

## 3 Data Description

The data published in this article expand on previously published data that described the development of a thermosensitive liposome formulation of the heat shock protein 90 inhibitor alvespimycin (ThermoALV), *in vitro* characterization of the combination effects of alvespimycin (ALV) and doxorubicin (DXR) in a panel of breast cancer cell lines, as well as the *in vivo* distribution of the drugs following administration in thermosensitive liposomes in combination with mild hyperthermia (HT) in MDA-MB-231 tumor bearing female SCID mice [1]. Here, the combination effects of DXR and ALV are characterized in the murine breast cancer cell line 4T1 under conditions of HT. In addition, the therapeutic efficacy and treatment associated toxicity of this thermosensitive liposomal combination are evaluated in both immunocompromised and immunocompetent mouse breast cancer models. These results were summarized into a dataset which has been published in the publicly accessible repository [2]. The dataset consists of one excel file with multiple spreadsheets which are named based on the data they include, specifically “4T1-Cell data”, “231-Tumor volume & body weight”, “231-Survival data”, 4T1-Tumor volume & body weight”, and “4T1-Survival data”.

### 3.1 Drug combination effects in 4T1 breast cancer cells

**Fig. 1:**
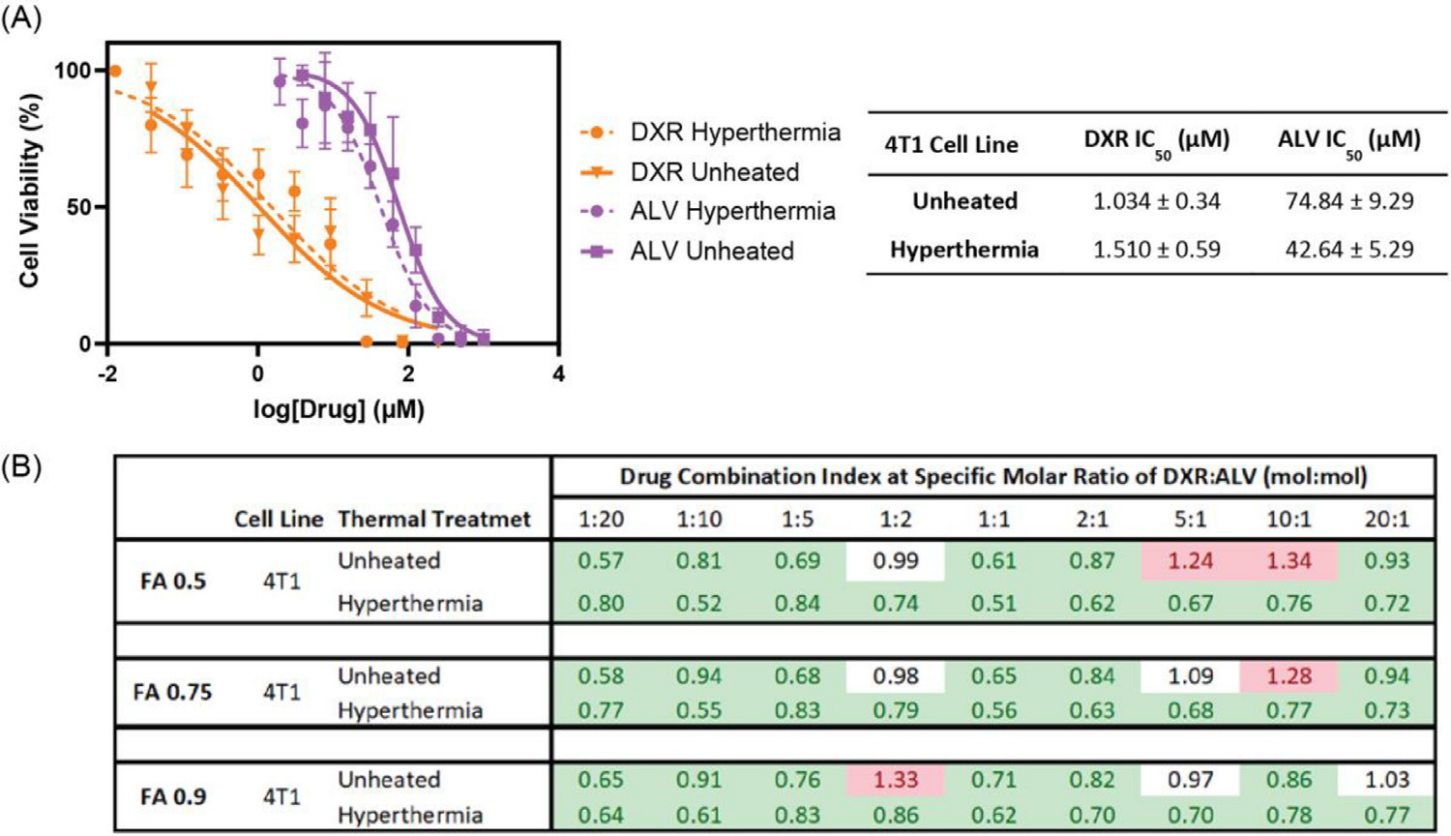
Cytotoxic effect of DXR and ALV in 4T1 cell line. (A) Cell viability following DXR or ALV treatment with or without hyperthermia (*n* ≥ 4). (B) The combination index (CI) values for DXR and ALV at different fractions affected (FA) in the 4T1 cell line (*n* ≥ 3). Synergistic, additive, and antagonistic combination effects are colored in green, white, and red, respectively. CI and FA results were calculated using CalcuSyn software which was developed based on the Chou-Talalay method.

The application of HT decreased the half maximal inhibitory concentration (IC_50_) of ALV by a factor of 1.76 (*p* > .09) but increased the DXR IC_50_ by 1.46-fold (*p* > .4). Additionally, the IC_50_ values of ALV at both temperatures were more than 25-fold higher than the IC_50_ values of DXR, indicating that 4T1 cells are much more sensitive to DXR treatment. The combination of DXR and ALV was shown to be synergistic in the 4T1 cell line at most of the conditions evaluated. DXR and ALV at molar ratios of 1:2, 5:1, 10:1, and 20:1 appeared to have additive or even antagonistic effects at 37 °C, but once HT was applied to the cells, the drug combination acted synergistically (Fig. 1).

### 3.2 Efficacy and toxicity of ThermoDXR and ThermoALV in combination with HT in female SCID mice bearing orthotopic MDA-MB-231 tumors

**Fig. 2:**
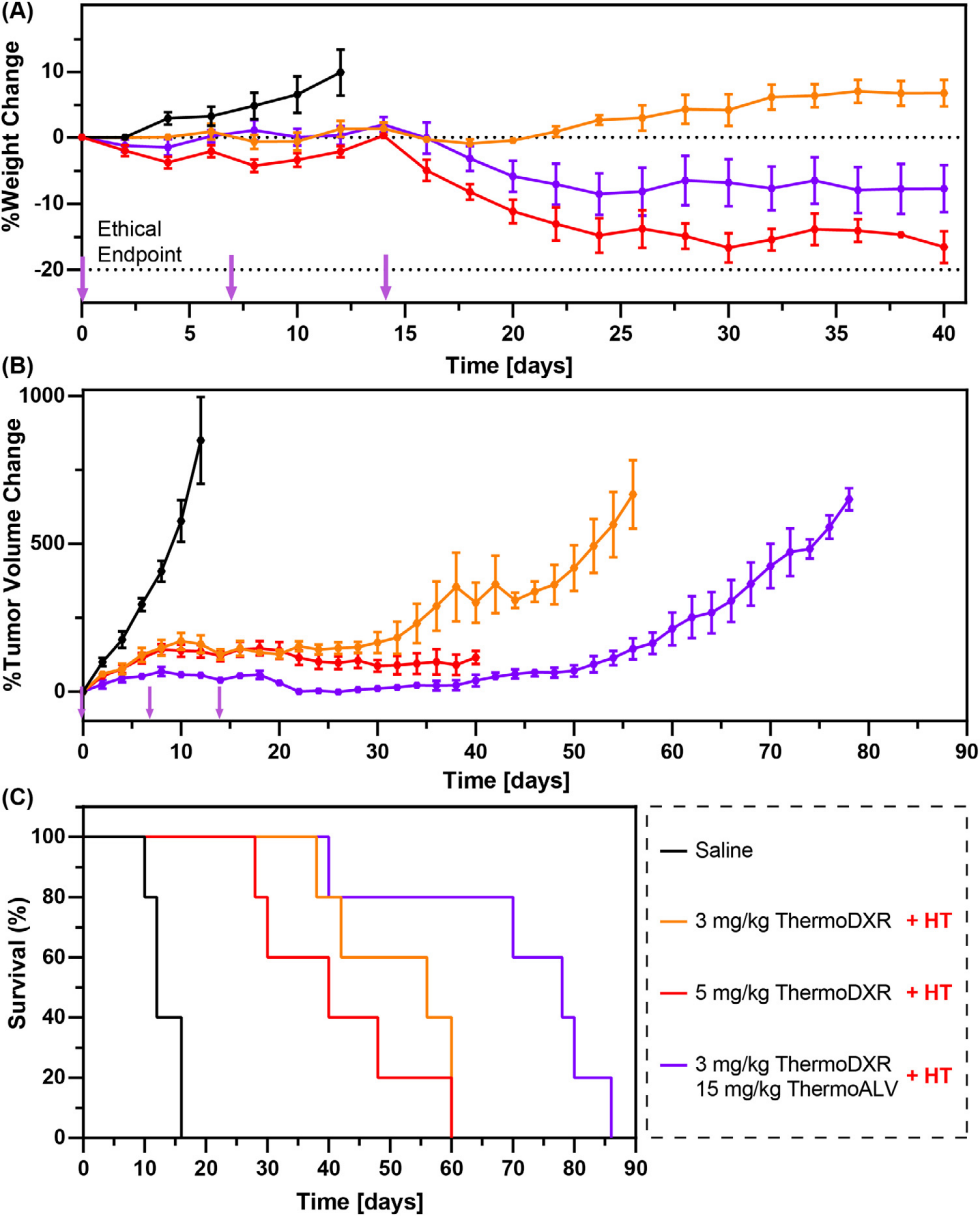
Female SCID mice bearing orthotopic MDA-MB-231 tumors received intravenous thermosensitive liposome formulations of drug in combination with localized mild hyperthermia on days 0, 7, and 14 (labeled with purple arrows). Tumors were heated using a laser-based heating system to a target temperature of 42.5 °C for five minutes followed by intravenous administration of liposomes and another 20 min of mild hyperthermic heating. Treatment groups included (i) thermosensitive liposomal doxorubicin (ThermoDXR) at 3 mg DXR/kg body weight; (ii) ThermoDXR at 5 mg DXR/kg body weight; or (iii) a combination of ThermoDXR at 3 mg DXR/kg body weight and a thermosensitive liposome formulation of the heat-shock protein 90 inhibitor alvespimycin (ThermoALV) at 15 mg ALV/kg body weight. Control animals received intravenous saline on the respective treatment days. Treatment associated toxicity was monitored in terms of body weight changes with animals that lost more than 20 % of the initial weight being euthanized. Treatment efficacy is shown in terms of tumor growth as well as overall animal survival. Data is presented as mean ± SEM (*n* ≥ 3).

Normalized animal weight (A) and tumor volumes (B) as well as overall survival (C) following treatment with ThermoDXR and ThermoALV in combination with HT in female SCID mice bearing MDA-MB-231 orthotopic tumors are presented in Fig. 2.

Treatment with high dose ThermoDXR resulted in a 16.6% body weight loss (on day 40 post treatment). Four out of five mice treated with high dose ThermoDXR reached their ethical endpoint due to weight loss exceeding 20% of their original body weight. Only animals treated with low dose ThermoDXR experienced weight gain, and the average body weight on day 40 was 6.7% higher than the initial measurement. The addition of ThermoALV to low dose ThermoDXR led to 9.7% weight loss during the 40-day time period, and no animals in this group experienced weight loss greater than 20% initial body weight (i.e., ethical endpoint).

Tumor growth of animals treated with low dose ThermoDXR was significantly reduced compared to controls (*p* < .001; treatment day 12). However, the time period of effective tumor growth delay was significantly shorter in this group compared to animals treated with high dose ThermoDXR. Specifically, the low dose ThermoDXR group experienced evident tumor growth starting from approximately day 30 post treatment, while the high dose ThermoDXR group showed signs of increasing tumor growth rate on approximately day 40 post treatment. It is important to note that tumor growth analysis of animals treated with high dose ThermoDXR was limited to a significantly shorter duration for the study given that the animals reached ethical endpoint (i.e., weight loss >20%). Combining low dose ThermoDXR and ThermoALV (15 mg ALV/kg body weight) provided greater tumor growth inhibition than low dose ThermoDXR alone. This led to an observable 1.4 and 2-fold increase in median survival time compared to treatment with low dose or high dose ThermoDXR, respectively (78 ± 9 days versus 56 ± 15 days and 40 ± 11 days). However, the differences between combination group and low/high dose ThermoDXR were not statistically significant (*p* > .08).

### 3.3 Efficacy and toxicity of ThermoDXR and ThermoALV in combination with HT in female BALB/c mice bearing orthotopic 4T1 tumors

**Fig. 3:**
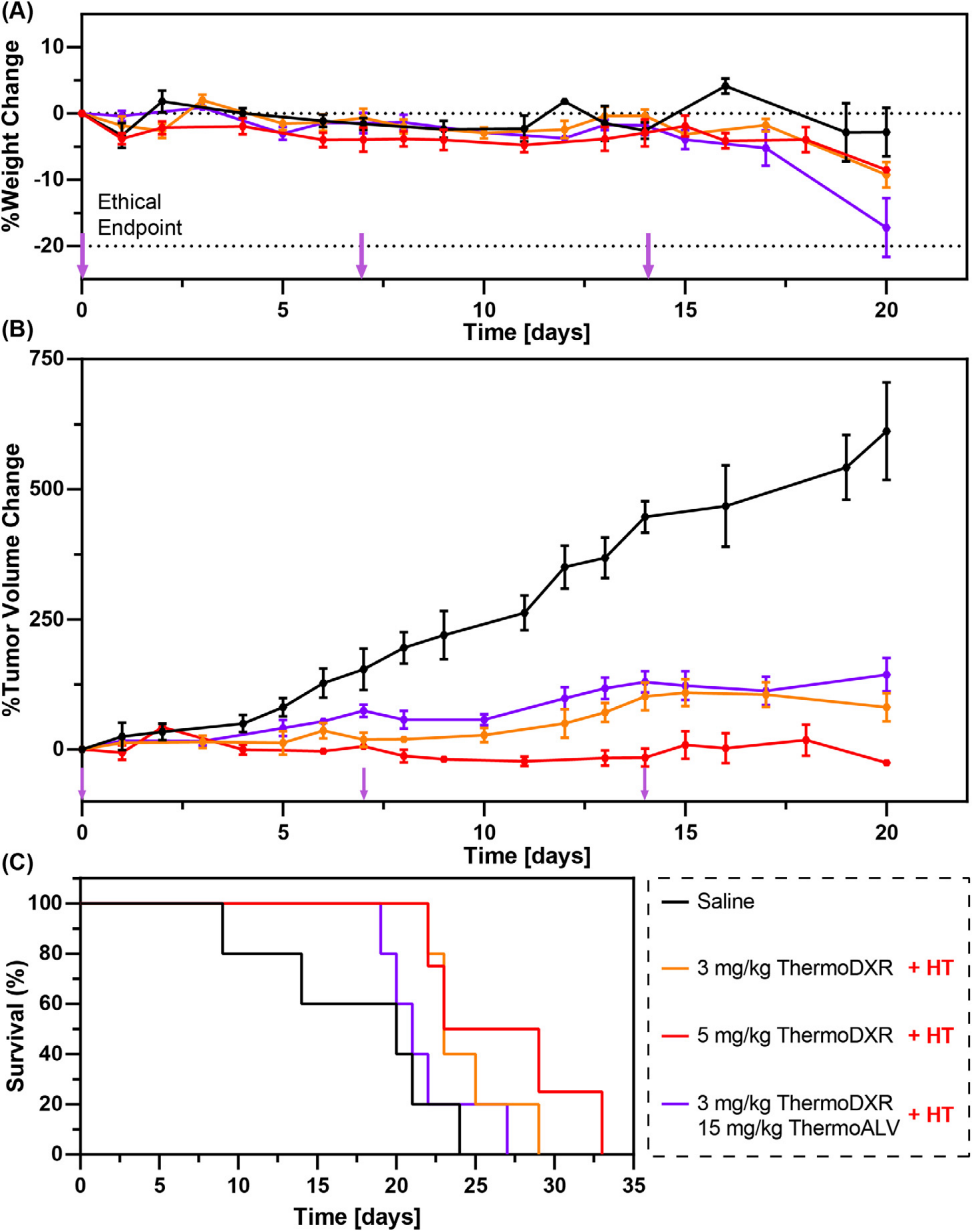
Female BALB/c mice with orthotopic 4T1 tumors received intravenous thermosensitive liposome formulations of drug in combination with localized mild hyperthermia (42.5 °C, 25 min) on treatment days 0, 7, and 14 (labeled using purple arrows). Treatment included low (3 mg DXR/kg body weight) and high doses (5 mg DXR/kg body weight) of thermosensitive liposomal doxorubicin (ThermoDXR) as well as low dose ThermoDXR in combination with a thermosensitive liposome formulation of the heat shock 90 protein inhibitor alvespimycin (ThermoALV at 15 mg ALV/kg body weight). Control animals received intravenous saline administration on each of the treatment days. Data is presented as mean ± SEM (*n* ≥ 3).

Normalized animal weight (A) and tumor volumes (B) as well as overall survival (C) following treatment with ThermoDXR and ThermoALV in combination with HT in female BALB/c mice bearing orthotopic 4T1 tumors are presented in Fig. 3. One mouse from the saline group did not receive all treatments as it was necessary to terminate before the third treatment due to excessive tumor volume.

The average weight change for all groups did not exceed 20% body weight loss during the observation period, with the exception of one mouse in the saline group and one mouse in the combination group which reached < 20% body weight on treatment day 20. During the initial treatment period (i.e., days 0 to 14) mice treated with high dose ThermoDXR lost more body weight than mice in the other groups, but quickly recovered shortly after treatment completion (day 15). The differences observed on day 20 of the study were not found to be statistically significant (*p* > .08).

Additionally, treatment with low/high dose ThermoDXR or the combination of ThermoDXR and ThermoALV resulted in comparable tumor volumes on day 20 of the study (*p* > .4), with a decrease in tumor volume of at least 3.6-fold for all treatment groups in comparison to the control (*p* < .001). The administration of thermosensitive liposomes in combination with HT moderately prolonged the median survival time (i.e., low dose ThermoDXR 23 ± 1 days, high dose ThermoDXR 26 ± 4 days, and combination 21 ± 1 days) in comparison to saline treatment (20 ± 7 days). Nonetheless, this did not result in statistically significant differences in median survival time (*p* > .2).

## 4 Experimental Design, Materials and Methods

### 4.1 Thermosensitive liposome formulation of doxorubicin (ThermoDXR)

Thermosensitive liposomal DXR was prepared as previously described by Dunne et al. [1]. Briefly, DPPC, lyso-SPC and PEG_2k_-DSPE at a molar ratio of 86/10/4 were dissolved and thoroughly mixed in chloroform followed by solvent removal using a rotary evaporator. The lipid film was then dried under a vacuum overnight to remove residual solvent. The lipids were subsequently hydrated for 30 min at 55 °C in sodium citrate buffer (300 mM, pH 4.0) to a lipid concentration of 125 mM. The rehydrated lipid mixture was then extruded three times through double-stacked 200 nm track-etch pore sized membranes at 55 °C and 200 psi nitrogen pressure using a 10 mL Lipex Extruder (Northern Lipids, Vancouver, Canada) followed by 10 times extrusion through double-stacked 100 nm pore sized membranes at 400 psi. Immediately after extrusion, the liposomes were chilled on ice for 10 min followed by storage at 4 °C until loading with DXR.

The external pH of the liposomes was raised to 7.4 by adding sodium carbonate buffer (0.5 M, pH 11.0) prior to loading with DXR. The liposomes were then pre-heated at 35 °C for 10 min in a temperature-controlled water bath. DXR (5 mg/mL in water) was added to the liposomes at a drug-to-lipid (D/L) ratio of 1 mg DXR per 20 mg lipid. The liposome drug mixture was then incubated for another 60 min at 35 °C while being gently stirred using a magnetic stir plate. The loaded liposomes were subsequently transferred into a glass vial and left to cool on ice for 10 min. Overnight dialysis (MWCO 50 kDA) in 200-fold excess HEPES buffered saline (HBS; 20 mM HEPES, 150 mM sodium chloride, pH 7.4) at 4 °C was performed to remove unencapsulated DXR. DXR loaded thermosensitive liposomes were concentrated using a tangential flow filtration system (Polysulfone MicroKros®, Spectrum, Rancho Dominguez, CA, USA), were filtered (0.22 µm PES filter) into a sterile rubber-sealed amber glass vial (ALK-Abelló Inc., Round Rock, TX, USA), and stored at 4 °C prior to administration to animals.

### 4.2 Thermosensitive liposome formulation of alvespimycin (ThermoALV)

Thermosensitive liposomal ALV (ThermoALV) was prepared as previously described (Dunne et al.) [1]. ThermoALV was designed with the same lipid composition as ThermoDXR with a goal towards achieving similar *in vivo* performance. Lipids were dissolved in chloroform followed by solvent evaporation to form a thin lipid film. Triethylamine sucrose octasulphate (TEA_8_SOS) was prepared as previously described [1]. Briefly, ion exchange chromatography (Dowex 50WX8-200) was used to exchange sodium ions of sodium sucrose octasulphate with protons. Subsequently, the eluate was titrated to a pH of 5.7 using neat triethylamine. Then, a solution of TEA_8_SOS was adjusted to a sulphate group concentration of 0.65 M. This TEA_8_SOS solution was then added to the thin lipid film at a lipid concentration of 100 mM. Following one hour of hydration at approximately 60 °C, liposomes were extruded as described in the previous section. TEA_8_SOS buffer exchange was achieved by overnight dialysis in 100 mM carbonate buffer (pH 10.0) at 4 °C. ALV was first dissolved in Milli-Q water at 10 mg/mL and then added to pre-warmed liposomes at a 1:20 drug-to-lipid weight ratio. ALV loading was performed at 35 °C for 30 min. Unencapsulated drug was removed by overnight dialysis in HBS buffer (pH 7.4) at 4 °C. Finally, ThermoALV was concentrated using tangential flow and filtered (0.22 µm PES filter) as described in Section 2.1.

### 4.3 Characterization of thermosensitive liposomes

Liposomes were characterized in terms of size, surface charge, and melting phase transition temperature (T_m_) prior to *in vivo* use. Liposome size was determined following 100-fold dilution in phosphate buffered saline (PBS, pH 7.4), using dynamic light scattering (Zetasizer Nano ZS, Malvern Instruments, Malvern, WOR, UK) and intensity-based analysis. The same system was used to determine the zeta potential after 100-fold dilution in Milli-Q water. A TA Q100 differential scanning calorimeter (TA Instruments, New Castle, DE, USA) was used to measure the T_m_ of the liposomes. Samples were heated and cooled from 25 °C to 60 °C for a total of three cycles at a rate of 1 °C/min.

DXR was quantified following a modified protocol by Needham et al. [3]. Briefly, a BioTek Cytation 5 plate reader (BioTek, Agilent Technologies, Santa Clara, CA, USA) set to excitation and emission wavelengths of 470 nm and 555 nm, respectively, was used to fluorometrically quantify DXR. Liposomes were diluted using HBS and lysed with 10% Triton X-100. Calibration samples with DXR concentrations ranging from 0.01 µg/mL to 2 µg/mL in HBS, containing the same amount of Triton X-100 as the liposome samples, were prepared.

ALV concentrations were determined by high-performance liquid chromatography-mass spectrometry (HPLC-MS) which included a 1260 Infinity HPLC system (Agilent, Mississauga, ON), an Agilent EC-C18 column (1.9 µm, 2.1 × 50 mm, Agilent, Mississauga, ON), and a TSQ Endura™ Triple Quadrupole MS (Thermo Fisher Scientific, Mississauga, ON). The mobile phase was composed of 0.1% formic acid and acetonitrile containing 0.1% formic acid at a ratio of 70:30. Standard solutions were prepared by dissolving ALV in methanol with concentrations ranging from 5 ng/mL to 1000 ng/mL.

### 4.4 Cell culture and evaluation of *in vitro* cytotoxicity

MDA-MB-231 cells were cultured in DMEM/F-12 (1:1) media and 4T1 cells in RPMI-1640 media containing 2 mM L-glutamine, 10 mM HEPES, 1 mM sodium pyruvate, 4500 mg/L glucose, and 1500 mg/L bicarbonate, respectively. Both media were supplemented with 10% FBS and 1% P/S and cells were kept at 37 °C and 5% CO_2_, unless indicated otherwise. 4T1 cells were seeded in 96 well plates at 1000 cells per well, followed by incubation for 24 hours. Subsequently, to evaluate the cytotoxicity of DXR and ALV alone, or to determine their combined effects at specific DXR:ALV molar drug ratios (i.e., 20:1, 10:1, 5:1, 2:1, 1:1, 1:2, 1:5, 1:10, 1:20), cells were incubated with drugs at different dilutions for one hour, followed by washing of cells twice with PBS. To determine the effect of mild hyperthermia the cells was incubated for one hour at 42 °C and 5% CO_2_. After a total incubation period of 48 h (i.e., post addition of drug to cells), cell viability was measured using a standard acid phosphatase assay. Specifically, cells were incubated with phosphatase substrate (4-nitrophenyl phosphate; 2 mg/mL) for 2 h, followed by addition of 0.1 N NaOH. UV absorbance was then measured at 405 nm. GraphPad Prism 6.0 (GraphPad Software, San Diego, CA, USA) was used to normalize the absorbance data with positive and negative controls and fit the data with a 4-parameter sigmoidal dose response curve to calculate IC_50_. CalcuSyn Version 2.0 was used to determine combination indices at different fractions affected (i.e., cell inhibition percentages). A combination index < 0.9 is indicative of a synergistic DXR:ALV effect, a value between 0.9 and 1.1 indicates additive effects, and > 1.1 indicates an antagonistic drug effect.

### 4.5 Breast cancer tumor models

All animal studies were conducted in accordance with the guidelines of the Animal Care Committee of the University Health Network (UHN, Toronto, ON, Canada). To establish orthotopic tumors, 1.2 × 10^6^ MDA-MB-231 cells suspended in Dulbecco's phosphate-buffered saline were injected into the lower right abdominal mammary fat pad of 6–8-week-old female SCID mice. Tumors were left to grow for approximately 12–14 days to reach a volume of > 120 mm³. Similarly, 1 × 10^5^ 4T1 cells were injected into the lower right abdominal mammary fat pad of 6–8-week-old female BALB/c mice. Animals were randomized to each experimental group ensuring a group size of five animals, except for the 5 mg/kg ThermoDXR group in the syngeneic 4T1 breast tumor study which contained four mice. Tumor volumes were determined using caliper measurements of tumor length (l) and width (w): $V=\;\frac{\pi }{6}\left(w^{2}\right)\left(l\right)$. Tumors measuring > 15 mm in any dimension, or body weight loss > 20 %, were selected as ethical endpoints. GraphPad Prism 6.0 was used to visualize data and to perform Kaplan-Meier survival analysis. % weight change and % tumor volume increase were then plotted as the mean ± SEM. Calculation of the mean required a minimum of three animals per group.

### 4.6 Chemotherapy and hyperthermia treatment

Chemotherapy treatment included: (i) ThermoDXR at 3 mg DXR/kg body weight (low dose), (ii) ThermoDXR at 5 mg DXR/kg body weight (high dose), or (iii) ThermoDXR at 3 mg DXR/kg body weight together with ThermoALV at 15 mg ALV/kg body weight. Control animals were treated with sterile 0.9 % saline solution. Treatments were administered via intravenous tail vein catheter injection on days 0, 7, and 14. Concurrently, localized mild hyperthermia was applied to tumors using a custom-built laser-based heating system [4]. In brief, an illuminator was placed over the tumor and a 400 µm fiber was used to deliver 763 nm laser light from the diode laser to the illuminator. A point-based optical fiber temperature probe (Luxtron Model 790, LumaSense Technologies Inc., Santa Clara, CA, USA) was used to monitor tumor temperatures. The laser power was adjusted to achieve a tumor temperature of 42.5 °C. Tumors were pre-heated for 5 min, followed by intravenous treatment administration and another 20 min of heating.

### 4.7 Statistical analysis

SSPS Statistics 28.0 (IBM, Armonk, NY, USA) was used for statistical analysis. Animal weights and tumor volumes were compared on specific treatment days by one-way ANOVA with Bonferroni *post hoc* correction. Log-rank analysis with Bonferroni correction for multiple comparisons was used to determine differences in animal survival.

## Ethics Statements

All animal studies were conducted in accordance with the guidelines of the Animal Care Committee of the University Health Network (UHN, Toronto, ON, Canada). The experiments complied with the ARRIVE guidelines and were performed in accordance with the National Institutes of Health guide for the care and use of laboratory animals (NIH Publications No. 8023, revised 1978).

Female mice were used to establish the MDA-MB-231 and 4T1 breast cancer models. Due to the predominance of breast cancer in females, employing female animals enables us to closely mimic the biological context and improve the translational value of our findings.

## Funding

This work was supported by the 10.13039/501100000024Canadian Institutes of Health Research (PJT 155905).

## CRediT authorship contribution statement

**Xuehan Wang:** Investigation, Formal analysis, Writing – original draft, Visualization. **Maximilian Regenold:** Investigation, Formal analysis, Writing – original draft, Visualization. **Michael Dunne:** Conceptualization, Investigation, Formal analysis, Writing – review & editing. **Pauric Bannigan:** Writing – review & editing. **Christine Allen:** Conceptualization, Writing – review & editing, Supervision, Funding acquisition.

## Data Availability

Data demonstrating the in vivo anti-tumor efficacy of thermosensitive liposome formulations of a drug combination in pre-clinical models of breast cancer (Original data) (Mendeley Data)
